# Pathophysiological Role of Purinergic P2X Receptors in Digestive System Diseases

**DOI:** 10.3389/fphys.2021.781069

**Published:** 2021-12-24

**Authors:** Qimin An, Gengyu Yue, Xiaoxu Yang, Jun Lou, Weixi Shan, Jianhong Ding, Zhe Jin, Yanxia Hu, Qian Du, Qiushi Liao, Rui Xie, Jingyu Xu

**Affiliations:** ^1^Department of Gastroenterology, Affiliated Hospital of Zunyi Medical University, Zunyi, China; ^2^The Collaborative Innovation Center of Tissue Damage Repair and Regeneration Medicine of Zunyi Medical University, Zunyi, China

**Keywords:** P2X receptors, extracellular ATP, physiological functions, digestive system diseases, inflammation, tumor

## Abstract

P2X receptors (P2XRs) are trimeric, non-selective cation channels activated by extracellular ATP and widely distributed in the digestive system. P2XRs have an important role in the physiological function of the digestive system, such as neurotransmission, ion transports, proliferation and apoptosis, muscle contraction, and relaxation. P2XRs can be involved in pain mechanisms both centrally and in the periphery and confirmed the association of P2XRs with visceral pain. In the periphery, ATP can be released as a result of tissue injury, visceral distension, or sympathetic activation and can excite nociceptive primary afferents by acting at homomeric P2X(3)R or heteromeric P2X(2/3)R. Thus, peripheral P2XRs, and homomeric P2X(3) and/or heteromeric P2X(2/3)R in particular, constitute attractive targets for analgesic drugs. Recently studies have shown that P2XRs have made significant advances in inflammation and cancer. P2X7R mediates NLRP3 inflammasome activation, cytokine and chemokine release, T lymphocyte survival and differentiation, transcription factor activation, and cell death. The P2X7R is a potent stimulant of inflammation and immunity and a promoter of cancer cell growth. This makes P2X7R an appealing target for anti-inflammatory and anti-cancer therapy. It is believed that with the further study of P2XRs and its subtypes, P2XRs and its specific antagonists will be expected to be widely used in the treatment of human digestive diseases in the future.

## Introduction

**Figure 1:**
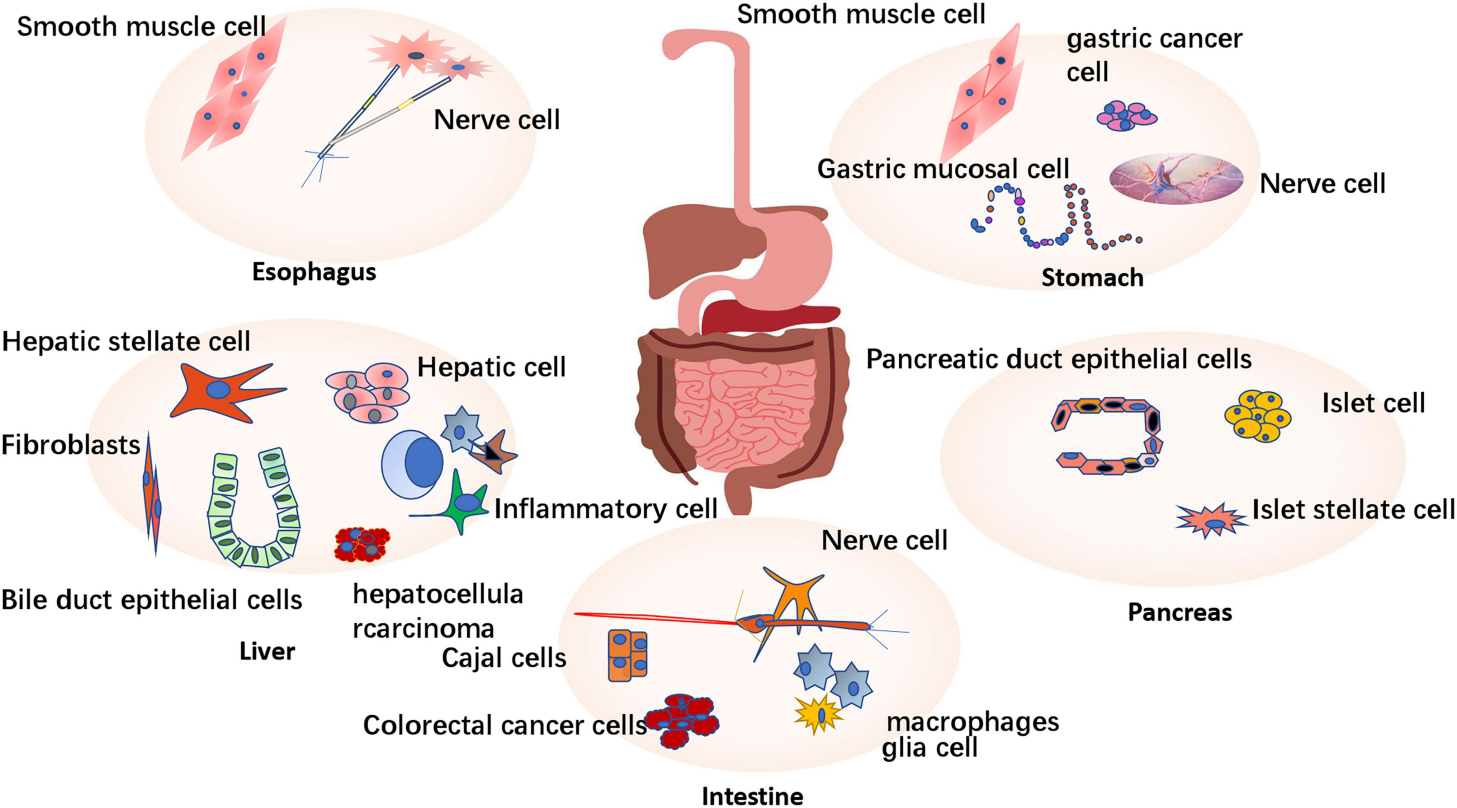
Expression and distribution of P2X receptor in digestive system. P2X receptors are widely expressed in the digestive system. P2X receptors are mainly expressed in the esophageal vagal afferent neurons and esophageal smooth muscle cells. The stomach is mainly expressed in submucosal nerve plexus, myenteric nerve plexus, smooth muscle cells, and gastric cancer cell lines. It is widely expressed in hepatic parenchyma cells and also expressed in some non-parenchymal cells (such as bile duct cells, liver fibroblasts, immune cells and fibroblasts, hepatic stellate cells, and hepatocellular carcinoma). It is expressed in pancreatic duct epithelial cells, islet cell lines, and pancreatic hepatic stellate cell lines. In the intestine, P2X receptors are mainly expressed in the submucosal nerve plexus, intermuscular nerve plexus, Cajal cells (ICC), intestinal immune cells (macrophages and glia cells), and colorectal cancer cells.

Purine signal was first discovered in 1972 (Burnstock, 1972). It was not until 1976 that Burnstock and his team first proposed the concept of purine receptor (Burnstock, 1976). Purine receptors can be divided into P1 receptors whose main ligands are adenosine and P2 receptors whose main ligands are nucleotides according to their ligand types. P2 receptors (P2Rs) fall into two classes, the ionotropic P2X receptors (P2XRs) and the metabotropic P2YRs (Burnstock, 2018). P2XRs are cell membrane cation channels that are gated by extracellular ATP, and the ATP-mediated opening of these channels allows Ca2+ and Na+ influx, as well as K+ efflux. Seven subtypes of P2X receptors have been cloned and denoted as P2X1 to P2X7, and in three heteromeric receptors identified as P2X2/3, P2X4/6, and P2X1/5, with functional channels assembled by homo- or heterotrimers (Saul et al., 2013). In 2012, Hattori et al. reported the open crystal structure of the zfP2X4 receptor with ATP in its binding site, which confirmed previous studies on ATP recognition and provided structural insight into the channel gating of P2X receptors (Hattori and Gouaux, 2012). Their research showed that All subtypes share a common topology containing two transmembrane domains (TM1and TM2), a large Cys-rich extracellular domain (~280 residues) and intracellular C- and N-termini. Because the overall structure of each receptor subunit resembles the shape of a “leaping dolphin,” thus different domains of P2X are named as head, dorsal fin (DF), left flipper (LF), right flipper (RF), body, and fluke. The TM helices (TM1 and TM2) of a single subunit delineate the “fluke” of the “dolphin” and are involved in many properties of P2X receptors, including unitary conductance and rectification, differential desensitization among subtypes, and voltage-dependence of P2X receptors. The extracellular domain delineate the “head” and “LF” or “DF” and “upper body domains” of “dolphin” harbors binding sites for ATP, competitive antagonists, and modulatory metal ions. The N-terminus is composed of approximately 30 amino acid residues, while the C-terminus comprises approximately 30–240 amino acid residues, varying among different subtypes (Hattori and Gouaux, 2012; Jiang et al., 2012, 2013; Habermacher et al., 2016; Wang and Yu, 2016). Therefore, these structural characteristics of P2X determine the gating process of P2X receptors. Following ATP binding, the head domain moves downward, the DF domain moves upward and the LF domain is pushed away from the binding site. Because of the coupling between the LF, DF, and lower body domains, the relative motions of the LF and DF are capable of driving the outward expansion of the lower body, followed by the movements of TMs and subsequent opening of the ion access route. In conclusion, the gating process consists of a series of complicated and coordinated motions of multiple domains, which leads to the final channel opening of P2X receptors (Wang and Yu, 2016). This conformational change makes the channel highly permeable to Na^+^, K^+^, and Ca^2+^, which triggers rapid membrane depolarization (Hattori and Gouaux, 2012; Kawate, 2017). A large number of studies have found that P2XR(s) are widely expressed in excitatory and non-excitatory cells, such as neuron, glia, platelet, epithelia, and macrophage (Figure 1), and participate in a series of important physiological and pathological processes, including the regulation of synaptic transmission, smooth muscle contraction, pain perception, inflammation, cardiovascular modulation, immunomodulation, and tumorigenesis. In this review, we mainly emphasize the important role of P2XR(s) in the physiology and pathology of the esophagus, stomach, liver, pancreas, and colon (Table 1).

**Figure 2:**
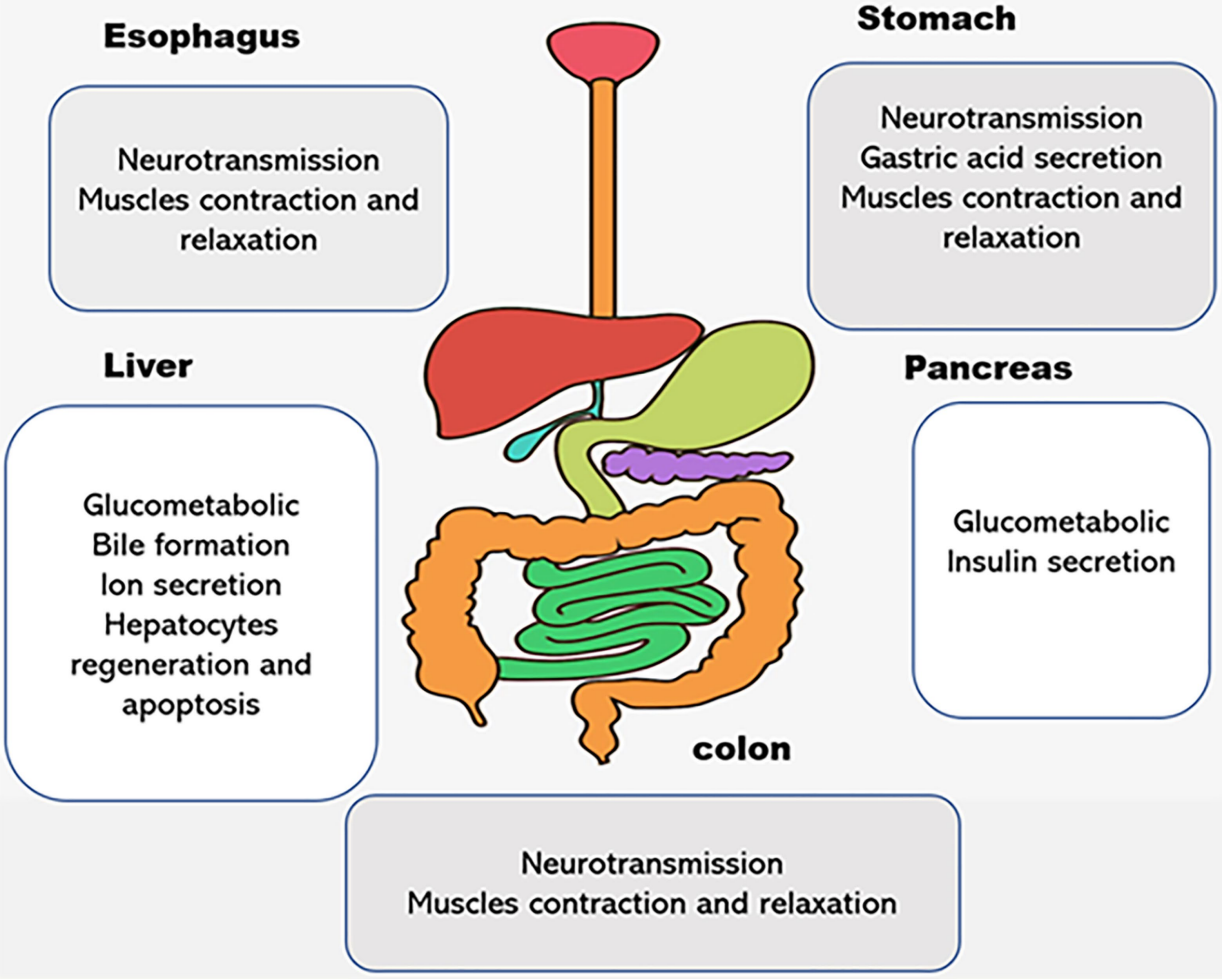
The physiological functions of P2X receptors in digestive system. Different subtypes of P2X receptors are expressed in human esophagus, stomach, liver, pancreas, and colon. They play different roles in the regulation of physiological processes, such as neurotransmission, ion transports, proliferation and apoptosis, muscle contraction, and relaxation in the digestive organs.

**Figure 3:**
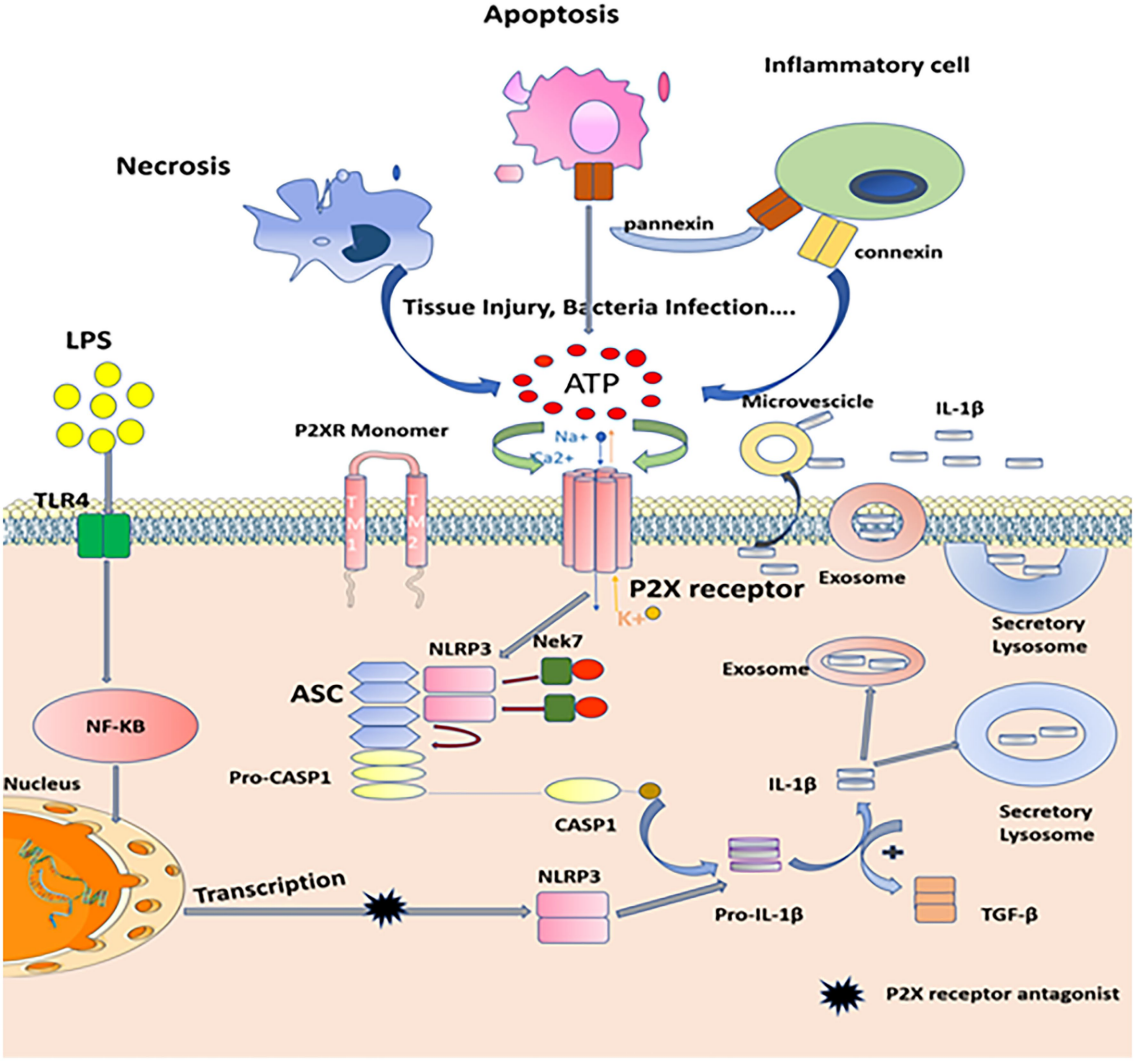
Mechanisms of P2X7 receptors in inflammatory processes. When tissue injury, infection, necrosis, apoptosis, or inflammatory cells are activated, many types of cells release nucleotides (such as ATP or ADP) from inside to outside and accumulate in large quantities. This process involves a variety of molecular pathways, such as pannexin-mediated ATP release during apoptosis and connexin or pannexin-mediated ATP release from inflammatory cells, such as neutrophils. Extracellular ATP acts as a signal molecule by activating P2X receptors, which induces conformational changes of P2X receptor and increases the plasma permeability of cell membrane to Na+, K+, and Ca2+. A large amount of K+ outflow triggers the assembly of NEK7, NLRP3, Asc, and caspase-1, catalyzes the cleavage of pro-IL-1β, matures Pro-IL-1β into IL-1β, and finally promotes inflammation and immune response through the release of secretory lysosomes, exocrine, and microvesicles. And the production of inflammatory factors, such as IL-1β, can promote the expression of α-smooth muscle actin (α-SMA) and TGF-β1. Besides, PAMPs (LPS) promote transcription of the genes encoding IL-1β and inflammasome components, such as NLRP3, this process can be blocked by P2X receptors antagonist.

**Table 1:** Expression and distribution of P2X receptor in digestive organs and its biological effect with diseases of digestive system.

Organs	P2XR subtypes	Target cells	Related diseases	Biological effect	References
Esophagus	P2X3R	Esophagus vagal primary afferent neurons	Esophagus hypersensitivity	P2X3R promotes esophagus hypersensitivity by indirectly enhancing afferent nerve mechanical sensitivity	Page et al., 2000; Banerjee et al., 2009; Miwa et al., 2010
Stomach	P2X3R	Nodose ganglion neurons	Dyspepsia hyperalgesia in gastric	Indirectly enhances signal transduction in the spinal cord and promotes dyspepsia and hyperalgesia in gastric	Dang et al., 2005; Blanke et al., 2019
	P2X7R	Gastric cancer cells	Gastric cancer	Enhances the proliferation, migration and invasion of gastric cancer cells, *via* modulating ERK1/2 and Akt pathways and EMT	Lili et al., 2019; Calik et al., 2020
Liver	P2X1R, P2X4R, P2X7R	Peripheral blood mononuclear cells (PBMC)	Viral hepatitis	Promotes PBMC-mediated immune response in chronic HCV infection	Manzoor et al., 2011, 2016; Ashraf et al., 2013
	P2X7R	Hepatocytes and macrophages	Alcohol-associated hepatitis	ATP-induced P2RX7 signaling and NLRP3 inflammasome produce IL-1β accelerated lipid accumulation in hepatocytes	Iracheta-Vellve et al., 2015; Li et al., 2018a,b; Freire et al., 2019
	P2X7R	Hepatocytes	Drug-induced liver injury	P2XR(s) ion channel opens a large number of Ca^2+^ influx. The balance of intracellular Ca^2+^ is disrupted, which aggravated the liver injury	Hoque et al., 2012; Amaral et al., 2013; Xie et al., 2013
	P2X7R	NKT cells	Autoimmune hepatitis	Activate or inhibit NKT cells to inhibit or promote autoimmune hepatitis	Kawamura et al., 2006; Beldi et al., 2008
	P2X4R, P2X7R	Kupffer cells, hepatic stellate cell	Liver fibrosis	Promote liver stellate cell activation, proliferation	LeRoy et al., 1990; Bataller and Brenner, 2005; Ferrari et al., 2006; Kawamura et al., 2006; Mariathasan and Monack, 2007; Rubartelli and Lotze, 2007; Bieghs and Trautwein, 2013; Huang et al., 2014; Gentile et al., 2015; Robert et al., 2016; Jiang et al., 2017
	P2X4R, P2X7R	Macrophages	Sepsis	P2X7R signaling on myeloid cells augments intracellular killing of bacteria in sepsis. P2X4R augment bacterial killing and protect against sepsis	Gu et al., 2000; Savio et al., 2017; Csóka et al., 2018; Larrouyet-Sarto et al., 2020
	P2X4R, P2X7R	Hepatocellular carcinoma cell	Liver cancer	Activate inflammasome, oxidative stress and immune regulation, promote tumor cell proliferation	Nakanishi et al., 2005; Ghalali et al., 2017
Pancreas	P2X7R	Pancreatic cells	Acute pancreatitis	P2X7R antagonist AME 439079 can reduce the pancreatic edema and significantly reduce pancreatic leukocyte infiltration	Hoque et al., 2011; Zhang et al., 2019
	P2X7R	Pancreatic stellate cells	Chronic pancreatitis	Regulates the activation, proliferation and apoptosis of pancreatic stellate cells	Künzli et al., 2008; Haanes et al., 2012; Wang et al., 2015
	P2X3R	DRG neurons		Pancreatic hyperalgesia in chronic pancreatitis	
	P2X7R	Islet β cells	Diabetes	Mediating insulin secretion, survival and apoptosis of islet β cells	Coutinho-Silva et al., 2003; Vieira et al., 2016
	P2X7R	Pancreatic ductal adenocarcinoma cell line	Pancreatic carcinoma	Regulates the survival, migration and invasion of pancreatic ductal adenocarcinoma cell	Mistafa and Stenius, 2009; Mohammed et al., 2012; Rodrigues et al., 2014; Giannuzzo et al., 2016
Colon	P2X2R, P2X3R, P2X5R, P2X6R	Colonic afferent nerve endings, Colon-specific dorsal root ganglion	Irritable bowel syndrome	Mediates mechanical sensory transduction such as visceral pain and promotes hypersensitivity	Wynn et al., 2003; Xu et al., 2008; Shinoda et al., 2009; Hu et al., 2017; Lashermes et al., 2018
	P2X4R, P2X4R, P2X7R	Intestinal immune cells (macrophages and T helper cells), Myenteric plexus	Inflammatory bowel disease	Promotes immune, inflammatory response, in dyskinesia and pain	Yiangou et al., 2001; Wynn et al., 2004; Volonté and Burnstock, 2013; Deiteren et al., 2015; Hofman et al., 2015
	P2X7R	Colorectal Cancer cells (HCT8 and Caco-2)	Intestinal cancer	Promotes the invasion and migration of colon cancer	Coutinho-Silva et al., 2005; Qian et al., 2017; Zhang et al., 2020

**Table 2:** Role of agonists and antagonists of the P2X receptors in digestive diseases.

P2XR	Regulator		Pharmacological function	References
P2X3R	Agonists	α,β-methylene-ATP	Promote esophageal hypersensitivity	Page et al., 2000
			Promotes gastric hypersensitivity	Kentish and Page, 2015; Burnstock, 2016a
			Promotes intestinal hypersensitivity	Giaroni et al., 2002
	Antagonists	A-317491	Blocks specifically P2X3R inhibition gastrointestinal tract hypersensitivity	Jarvis et al., 2002
		AZ004		Gever et al., 2010
		Diaminopyrimidines		Ballini et al., 2011
		AF-353		Ford, 2012
P2X4R	Agonists	e-ATP	Sustain hepatic myofibroblasts activated and fibrogenic phenotype	Huang et al., 2014
		Ivermectin	Against bacterial dissemination and mortality in sepsis	Benjamim et al., 2004
	Antagonists		–	–
P2X7R	Agonists	e-ATP BzATP	Contribute to the symptoms of IBD, including motor abnormalities, diarrheal state, and visceral pain	Lashermes et al., 2018
	Antagonists	A804598	Inhibits liver inflammation caused by combined chronic alcohol and high-fat diet	Ashraf et al., 2013
		A-438079	Promotes liver injury in acetaminophen hepatotoxicity	Li et al., 2018b
		A438079, BBG	Inhibits liver fibrosis	Mariathasan and Monack, 2007; Gentile et al., 2015
		A-439079	Reduce the pancreatic edema and significantly reduce pancreatic leukocyte infiltration, limit the Progression of pancreatitis	Burnstock, 2014
		AZ10606120	Inhibit the proliferation of pancreatic ductal adenocarcinoma	Novak et al., 2010

## Esophagus

In the past, it has been found that P2XR(s) are expressed and distributed in the esophagus, such as esophageal vagal afferent neurons and esophageal smooth muscle cells, and play an important role in the physiology and pathology of the esophagus. Intraganglionic laminar endings (IGLEs) are the transduction sites of the mechanical receptors of the vagus nerve in the esophageal muscle layer. It has been found that P2X2R, P2X3R, and isomeric P2X2/3R are expressed on the IGLEs in rats, mice, and guinea pigs, and the number of receptors in the ventral segment of the esophagus increases as they travel to the esophagus. Therefore, P2XR(s) may be an important regulator of the mechanical sensory characteristics of the esophageal vagus nerve (Castelucci et al., 2003; Wang and Neuhuber, 2003; Zagorodnyuk et al., 2003; Kestler et al., 2009). In addition, P2XR(s) are also expressed in the esophageal sphincter and smooth muscle. P2XR(s) can stimulate excitatory motor neurons on the esophageal sphincter to induce esophageal sphincter contraction in porcine lower esophageal sphincter (LOS; Lecea et al., 2009). In cat esophageal smooth muscle, α, β-metATP acts on P2XR(s) in a concentration-dependent manner to induce voltage-gated Ca^2+^ channel sensitive to dihydropyridine, so as to mediate Ca^2+^ influx and enhance EFS-induced contraction of esophageal smooth muscle induced by electric field (Cho et al., 2010) (Figure 2).

### P2X Receptors and Esophageal Pathology

Studies have shown that P2X3R is mainly expressed in nociceptive sensory neurons and plays a key role in nociceptive signaling (Wirkner et al., 2007). It has been documented that visceral afferent fibers undergo sensitization following visceral inflammation. For example, vagal mechanosensitive afferents innervating the esophagus of ferrets exhibit significantly greater response to P2X3 receptor agonist α,β-methylene ATP following esophagitis (Page et al., 2000). In 2009, Banerjee and his team found that in rats with a model of esophagitis, the expression of P2X_3_R was significantly upregulated in the vagus nerve and spinal afferent nerve, which may be related to the enhancement of esophageal hypersensitivity caused by esophagitis (Banerjee et al., 2009). Moreover, P2XR(s) indirectly enhances esophageal hypersensitivity by inducing changes in afferent nerve function, which is related to non-erosive reflux disease (NERD; Page et al., 2000; Miwa et al., 2010). Therefore, ATP and P2X3 ion channel receptor antagonists may be potential targets for the treatment of esophageal hypersensitivity (Table 2). In recent years, P2X3 receptor antagonists have been reported, such as A-317491, Diaminopyrimidines, AZ004 (Jarvis et al., 2002; Gever et al., 2010; Ballini et al., 2011; Ford, 2012).

## Stomach

It has been found that P2XR(s) are expressed in different parts of the stomach, such as Gastric smooth muscle cells, nodose ganglion cells, gastric vagal afferent nerve cells, gastric epithelial cells and gastric cancer cell lines, regulating gastric smooth muscle contraction, relaxation, nociceptive visceral sensory afferent, gastric acid secretion and invasion, and metastasis of gastric cancer. There are some differences on the role of P2XR(s) in regulating the contraction or relaxation of gastric smooth muscle, the earliest studies have found that P2XR(s) are involved in mediating gastric smooth muscle relaxation in rat and guinea pig gastric smooth muscle (Lefebvre and Burnstock, 1990; Matharu and Hollingsworth, 1992; Ahn et al., 1995). However, later studies have shown that P2XR(s) mediate gastric smooth muscle contraction. In 2005, Mule and his team confirmed that P2XR(s) are expressed on gastric excitatory neurons in mice, and gastric smooth muscle contraction is mediated by activation of ATP and α,β-meATP (Mule et al., 2005). The nodose ganglion (NG) is the main parasympathetic ganglion conveying sensory signals to the CNS from numerous visceral organs including digestive signals, such as gastric distension or the release the gastrointestinal peptides. The primary viscerosensory afferents of the vagus are comprised of a combination of unmyelinated C-type and lightly myelinated Aδ-type axons, and the cell bodies for these afferents reside in the nodose ganglion (NG; Gebhart and Bielefeldt, 2016). Vagal afferents relay stimuli from multiple visceral tissues including the heart, lungs, vasculature, gastrointestinal (GI) tract, and the accessory organs necessary for digestion. These primary visceral afferents must respond to organ specific stimuli in order to generate appropriate efferent responses. For example, vagal afferents innervating gastrointestinal (GI) tract chemical and mechanical stimuli to the brainstem as part of the parasympathetic control of nutrient homeostasis (Kentish and Page, 2015). ATP is a major diffusible signaling molecule that is released by neurons, glia, and immune cells, and is responsible for the proper control of GI functions and sensory afferents (Burnstock, 2016a). In addition, P2XR(s) expressed in the sensory afferent nerves and epithelial cells of the stomach can act as indirect acid receptors by increasing ATP release and activating P2XR(s) when extracellular pH is decreased, causing proton currents, thereby regulating gastric acid secretion (Holzer, 2011).

### P2X Receptors and Visceral Nerve Sensory Transmission

Blanke and his team confirmed that P2X3R is expressed in NG neurons and is sensitized following Ca^2+^ entry, thus causing neuroplastic changes within the NG neurons (Blanke et al., 2019). It has been found that when knockout of P2X3R gene slows the expansion of gastric vagal afferent nerve to stomach, considering the role of vagus nerve in the afferent signals of satiety and nausea, P2XR knockout may lead to the occurrence and development of dyspepsia. Therefore, P2X3R agonist may be a therapeutic target for gastrointestinal dyspepsia (McIlwrath et al., 2009). Dang and colleagues demonstrated this in a rat model of gastric ulcers, gastric sensory afferent neurons are overexcited and enhanced P2XR(s) activation, indirectly enhance signal transduction in the spinal cord, mediate gastric hypersensitivity, and promoted hyperalgesia in gastric ulcer under the influence of nociceptive stimuli, such as inflammation (Dang et al., 2005).

### P2X Receptors and Gastric Cancer

In recent years, some studies have found that P2X7R also plays an important role in the development and prognosis of gastric cancer. Lili and his team confirmed that P2X7R is overexpressed in gastric cancer cell lines and tissues, and is related to several malignant characteristics of gastric cancer, such as promoting the proliferation, migration, and invasion of gastric cancer cells by regulating ERK1/2, Akt pathway, and EMT. Therefore, P2X7R can be used as a biomarker of gastric cancer, which is helpful to the prognosis of gastric cancer (Lili et al., 2019; Calik et al., 2020).

## Liver

P2X receptors are widely expressed in the liver, including P2X1~P2X7 subtypes, P2X1R, P2X2R, P2X3R, P2X4R, and P2X7R are expressed in hepatocytes and Non-parenchymal cells, including hepatocytes, Kupffer cells, bile duct cells, hepatic stellate cell, hepatic fibroblasts, hepatocellular adenocarcinoma, and hepatocellular carcinoma (Fausther et al., 2012). Some studies have found that hepatocytes release ATP into the extracellular, these extracellular ATP can act as autocrine or paracrine signal molecules to regulate liver function by activating purinergic receptors on the plasma membrane. Earlier studies have found that extracellular ATP and UTP nucleotides infusion into the liver can stimulate glycogen decomposition and glucose release in rat hepatocytes (Buxton et al., 1986). In later, Emmett and his team found that in human Huh7 cells expressing P2X4R and P2X7R, BzATP, a selective agonist of P2XR(s), rapidly reduce the content of glycogen in Huh7 cells in the liver, which confirm that the activation of P2XR(s) could lead to glycogen decomposition, and P2XR(s) also play an important role in the glycogen decomposition of ATP. Moreover, the inactivation of glucose-activated transcription factor (ChREBP) in glycogen-rich hepatocytes leads to increased expression of P2X4R mRNA, which also suggests a functional relationship between P2XR(s) and glucose metabolism (Emmett et al., 2008). In addition, it was found that P2XR(s) regulate bile formation in hepatocytes and bile duct cells by relying on extracellular ATP. P2X4R is a key regulator of chloride current in bile formation and alkalization induced by ATP in mouse bile duct epithelial cells. ATP is released from cholangiocytes into bile and is a potent secretagogue by increasing intracellular Ca^2+^ and stimulating fluid and electrolyte secretion *via* binding purinergic P2 receptors on the apical membrane (Woo et al., 2010). In their review, Fausther and his colleagues mentioned that P2XR(s) activation can regulate ionic secretion in hepatocytes and cholangiocytes. For example, in murine and human hepatocytes, activation of P2X4R and P2X7R leads to increased Ca^2+^ and Na^+^ influxes, in rat hepatoma HTC cells, P2X4R activation modulates cellular regulatory volume decrease response by controlling the opening of volume-sensitive rectifying outwardly Cl^−^ channels. In Mz-Cha-1 bile duct cells, the addition of Cu~^(2+)^ to extracellular medium could inhibit Cl^−^-current induced by BzATP, indicating that P2XR(s) are also involved in ion secretion of bile duct cells (Fausther et al., 2012). In addition, in the mechanism of regulating liver regeneration and apoptosis, Gonzales and his team proposed that hepatocytes and Kupffer cells release large amounts of extracellular ATP, and promote liver regeneration after partial hepatectomy in rats. Interestingly, P2X4R was widely expressed in hepatocytes and Kupffer cells, mainly in the subtubule region closely related to lysosomal labeling. After partial hepatectomy, limited hepatocyte regeneration is observed in P2X4R knockout mice, accompanied by hepatocyte necrosis and cholestasis, impaired bile adaptability, changes in bile composition, and decreased release of adenosine triphosphate, and lysosomal enzymes. It is speculated that P2X4R purinergic signal contributes to the control of bile homeostasis through mechanisms involving peritubular lysosomes, thus protecting the liver from bile injury and hepatocyte proliferation after partial resection (Gonzales et al., 2010; Besnard et al., 2016). Interleukin-22 (IL-22) is released by immune cells and mediates strong hepatoprotective functions. *In vivo*-specific inhibition of P2X1 was associated with decreased IL-22 secretion, elevated liver injury, and impaired liver regeneration (Kudira et al., 2016). More interestingly, the study also found that P2X7R is involved in the regulation of programmed cell death in hepatocytes and NKT cells. The intracellular calcium concentration was increased by extracellular ATP, which mediates calcium-dependent cell death (Zoetewij et al., 1996). It is reported that high concentrations of BzATP (P2X7R agonist) and extracellular ATP can stimulate P2X7R to induce a large amount of Ca^2+^ influx and lead to apoptosis in rat hepatocytes *in vitro* (Gonzales et al., 2007). Therefore, we concluded that P2XR(s) play a key role in regulating liver glucose metabolism, bile formation, ion secretion, hepatocyte regeneration, and apoptosis. At the same time, P2XR(s) are also expressed in liver immune cells; thus, the study of P2XR(s) in liver immunology and inflammation has received great attention, although P2X1R and P2X4R seem to be involved in this (Ulmann et al., 2010; Fiebich et al., 2014), the role of P2X7R in inflammation has been best studied, probably because almost all congenital and adaptive immune cells express it (Monif et al., 2010; Di Virgilio et al., 2017). Adenosine triphosphate (ATP) is secreted from hepatocytes under physiological conditions and plays an important role in liver biology by activating P2 receptors. In contrast, injury or infection induces the release of ATP from cells, which triggers inflammation by activating P2X7R on immune cells to induce the release of inflammatory cytokines (Burnstock, 2016b). Therefore, the P2XR(s) act as a danger signal and enhance the immune response, such as viral hepatitis, alcoholic hepatitis, drug-induced hepatitis, autoimmune hepatitis, liver fibrosis, and liver cancer.

### P2X Receptors and Viral Hepatitis

To explore the mechanism of hepatitis C virus-induced liver pathogenesis in chronic hepatitis C, Ashraf and his team showed that the expression of ionic purine P2XR(s) on peripheral blood mononuclear cells (PBMC) of HCV patients is increased, mainly P2X1R and P2X7R, confirming the important participation of P2XR(s) in PBMC-mediated immune response in chronic HCV infection (Ashraf et al., 2013). Another study also confirmed that P2XR(s) also exist in human hepatocytes (Huh-7 cell line), in which P2X4R reacts to HCV structural protein E1E2, suggesting that P2X4R may be involved in the pathological process of HCV (Manzoor et al., 2011, 2016).

### P2X Receptors and Alcohol-Associated Hepatitis

In alcoholic steatohepatitis, mice exposed to alcohol directly lead to an increase in serum and extracellular ATP, inducing inflammasome activation and IL-1β to produce damage to liver cells. Interestingly, liver steatosis was attenuated in P2X7R knockout mice (Iracheta-Vellve et al., 2015). Freire and his team used C57BL/6 J mice to be exposed to a chronic mixture of ethanol and high-fat diet. Treatment with P2X7R antagonist A804598 reverses the changes in microglia and astrocytes and reduced mRNA levels of inflammatory markers (including IL-1β, iNOS, and CXCR2) and inflammatory signal transduction pathways (such as TLR2, CASP1, NF-kB1, and CREB1), as well as protein levels of Pro-IL-1β and Nf-kB1. It is suggested that P2X7R antagonist A804598 has a protective effect on inflammatory liver injury induced by chronic ethanol and high-fat diet (Freire et al., 2019). In addition, it was found that gentiopicroside ameliorates have a protective effect on alcoholic fatty liver induced by acetaldehyde in acute and chronic alcoholic fatty liver model, and could inhibit the activation of NLRP3 inflammasome thereby inhibiting the production of IL-1β and Caspase-1 by inhibiting the P2X7R (Li et al., 2018b). Li and his colleagues also found that Pleurotus citrinopileatus aqueous extract inhibits inflammation by regulating SIRT1-AMPK sum to improve alcoholic hepatic steatosis and inhibit the activation of P2X7R-NLRP3 inflammatory bodies, thus achieve the purpose of treating alcoholic fatty liver (Li et al., 2018a).

### P2X Receptors and Drug-Induced Liver Injury

In the mouse liver inflammation model induced by acetaminophen (APAP), excessive APAP can lead to liver injury and even hepatocyte necrosis, the impairment of dynamic balance of intracellular calcium (Ca^2+^) is a sign of hepatocyte toxicity induced by acetaminophen (APAP). The report of Amaral and his team showed that extracellular ATP produces and activates P2XR(s) in the process of hepatocyte injury induced by APAP. P2XR(s) ion channel opens a large number of Ca^2+^ influx. The balance of intracellular Ca^2+^ is disrupted, which aggravated the liver injury. P2XR(s) antagonist significantly eliminates the increase of intracellular calcium signal mediated by ATP (Amaral et al., 2013). In addition, in the process of liver injury and necrosis, increases extracellular ATP-activated P2X7R can also promote immune response and inflammatory response leading to liver caspase-1 and neutrophil migration to the liver, and activate the inflammatory corpuscular NLRP3 to release and process inflammatory factors, such as IL-1β, thus aggravating liver injury (Hoque et al., 2012). However, other studies have reported that P2X7R’s antagonist A-438079 protects the liver by affecting metabolism not by inflammasome activation, reduces glutathione GSH consumption by inhibiting P450 isoenzymes, reduces liver and mitochondrial protein adducts, reduces JNK activation, and promotes mitochondrial P-JNK translocation, oxidative stress, and liver injury (Xie et al., 2013).

### P2X Receptors and Autoimmune Hepatitis

It has been found that CD39 and P2XR(s) are expressed on NKT cells in mouse liver. CD39 regulates the production of nucleotide-mediated cytokines and limits their apoptosis through liver NKT cells, while autoimmune hepatitis induced by concanavalin A(Con A) is mainly liver NKT cell-mediated, Beldi and his team’s experimental results show that CD39 knockout is related to P2XR(s) activation and NKT cell apoptosis in Con A-induced autoimmune hepatitis. Knockout of CD39 has protective effect on autoimmune hepatitis induced by Con A (Beldi et al., 2008). However, another study reported that there are two situations in which P2X7R regulates NKT cells in autoimmune hepatitis. The binding of P2X7R to NKT cells inhibits the immature cells of NKT cells and stimulates activated cells. Therefore, P2X7R can inhibit or promote autoimmune hepatitis (Kawamura et al., 2006).

### P2X Receptors and Liver Fibrosis

P2X receptor(s) also play an important role in liver fibrosis. Liver fibrosis is a wound healing response to liver injury induced by many factors, including hepatitis virus infection, alcohol, autoimmune diseases, or drugs. During chronic liver injury, resting hepatic stellate cells are activated into myofibroblast-like phenotypes, which are characterized by high expression of smooth muscle α-actin (α-SMA) and deposition of large amounts of extracellular matrix, including type I collagen. Activated hepatic stellate cells produce inflammatory cytokines or chemokines under the action of interleukin-1β, tumor necrosis factor-α, or transforming growth factor-β signals. These inflammatory factors can promote the synthesis of extracellular matrix, promote cell proliferation, and lead to liver fibrosis (Bataller and Brenner, 2005; Bieghs and Trautwein, 2013; Robert et al., 2016). In previous reports, P2X7R is described as a “risk sensor,” which plays a key role in the release of danger signal ATP and drives inflammatory effectors activated by bacterial products to secrete inflammatory cytokines (Ferrari et al., 2006). In most cases, the elimination of pathogens requires damage-related molecular patterns (DAMP), including harmful endogenous cellular molecules released by necrotic cells (Rubartelli and Lotze, 2007). Extracellular ATP is an effective DAMP molecule, which induces the accumulation of NALP-3 inflammatory bodies by activating P2X7R and reducing intracellular K^+^. NALP-3 activates caspase-1 to induce pre-interleukin-1 (Pro-IL-1) maturation. IL-1 may upregulate different signal pathways through autocrine and paracrine signals, resulting in the increase of fibrogenic transforming factor-β1 (TGF-β1), the central mediator of fibrosis response in various tissues (Figure 3). This leads to fibrosis (LeRoy et al., 1990; Mariathasan and Monack, 2007; Gentile et al., 2015). From the study of the role of P2X7R in the activation of hepatic stellate cells by Jiang and his colleagues, it was found that the expression of Caspase-1mRNA, NLRP3mRNA-like receptor family (NLRP3mRNA) β, IL-1mRNA, IL-18, IL-6mRNA, and P2X7R is increased after LX-2 cells were directly treated with lipopolysaccharide (lps), and all of them are inhibited by P2X7R antagonist. Therefore, it is concluded that the activation of NLRP3 inflammatory bodies mediated by P2X7R may be involved in the production of IL-1β in hepatic stellate cells, and blocking the axis of P2X7R-NLRP3 inflammatory bodies may be a potential target for the treatment of hepatic fibrosis (Jiang et al., 2017). Huang and his team used an antagonist (A438079) to inhibit P2X7R to block collagen deposition and significantly reduce the expression of α-smooth muscle actin (α-SMA) and TGF-β1 (Huang et al., 2014). Tung and his colleagues also showed that P2X7R antagonist brilliant blue (BBG) downregulates TGF-β signal pathway and significantly decreases the expression of pro-inflammatory cytokines IL-6, IL-1β, and TNF-α in cirrhotic rats induced by choledochal ligation, and suggested that blocking P2X7R may be a therapeutic target for liver fibrosis and its complications (Tung et al., 2015). These reports confirmed the important role of P2X7R in liver fibrosis. In 2020, Jiang et al. explored the role of P2X7R in hepatic fibrosis induced by thioacetamide (TAA) and the mechanism of macrophage P2X7R promoting fibrosis. It was found that A438079, an antagonist of P2X7R, could reverse TAA-induced liver injury and fibrosis by antagonizing the activation of P2X7R-TLR4-NLRP3 axis (Jiang et al., 2020). Interestingly, in alcoholic liver fibrosis, P2X7R mediates acetaldehyde-induced activation of hepatic stellate cells through PKC-GSK3β signaling pathway, which promotes protein kinase C (PKC)/glycogen synthase kinase-3 β (GSK3 β)-dependent HSC proliferation and collagen production. P2X7R inhibitors may be a new drug for the treatment of alcoholic liver fibrosis (Wu et al., 2017). In addition, excepting P2X7R and P2X4R have also been reported to play a role in hepatic fibrosis. Studies by Le Guilcher and his team have found that increased expression of P2X4R stimulates calcium entry and lysosomal exocytosis, thereby releasing ATP and activating P2X4R-mediated signals by regulating cell contraction, adhesion and migration, and driving (HMF) activation in liver fibroblasts by controlling matrix synthesis degradation balance. As a result, the accumulation of extracellular matrix induces liver fibrosis, which is significantly attenuated when the P2X4R gene is knocked out or is inhibited by pharmacology (Le Guilcher et al., 2018). Therefore, P2XR(s) play a key role in the occurrence and development of liver fibrosis and provide a new target and direction for the treatment of liver fibrosis.

### P2X Receptors and Sepsis

Sepsis is a life-threatening organ dysfunction caused by a dysregulated host response to infection (Singer et al., 2016). Sepsis is mainly caused by inappropriate regulation of the immune system (Oberholzer et al., 2001; Benjamim et al., 2004), this dysregulation manifests as an inability to control bacterial growth and dissemination and by excessive inflammation, processes that are interrelated and are caused, in large part, by macrophage dysfunction (Csóka et al., 2015). In addition, macrophages are the primary P2X7R-expressing cell type (Gu et al., 2000). Csóka and his team found increased ATP levels in the plasma of septic mice, indicating increased extracellular ATP release, their results indicate that P2X7R signaling on myeloid cells augments intracellular killing of bacteria in sepsis (Csóka et al., 2015). Except for the P2X7R, Csóka and his team in a later study found Macrophage P2X4R augment bacterial killing and protect against sepsis, pharmacological targeting of P2X4R with the allosteric activator ivermectin protects against bacterial dissemination and mortality in sepsis (Csóka et al., 2018). The severity of sepsis can be linked to excessive inflammatory responses resulting in hepatic injury, P2X7R activation by e-ATP exacerbates inflammation by augmenting cytokine production, while CD39 (ENTPD1) scavenges e-ATP to generate adenosine, thereby limiting P2X7 activation and resulting in A2A receptor stimulation (Savio et al., 2017). Savio and his team found that CD39 expression in macrophages limits ATP-P2X7R-mediated pro-inflammatory responses. Moreover, the combination of P2X7 blockade with an adenosine A2A receptor agonist (ATL146e) completely protects the liver during sepsis, improving experimental outcomes (Savio et al., 2017). Larrouyet-Sarto et al. confirmed that P2X7 receptor expression increases in the liver of septic, when the P2X7R genetic deletion attenuates liver injury in septic mice and reduces oxidative stress induced by hepatic septicaemia in mice and reduces the number of inflammatory cells in liver tissue from septic mice. Therefore, these findings suggest possible administration of P2X7 receptor blockers to limit oxidative damage, inflammation, and liver injury during the acute phase of sepsis (Larrouyet-Sarto et al., 2020).

### P2X Receptors and Liver Cancer

In the study of Asif and his team, it was found that the expression of P2X4R and P2X7R is significantly increased in human hepatocellular carcinoma and hepatocellular adenocarcinoma compared with the control group. It is concluded that P2X4R and P2X7R are closely related to the downstream process of inflammation by activating inflammatory bodies, oxidative stress, and immune regulation, thus promoting the sustainable development of cancer and speculating that these two receptors are closely related to the quality of the tumor (Asif et al., 2019). Previous studies have shown that PI3K/Akt signaling pathway can be activated in hepatocellular carcinoma, and the activation of Akt is closely related to the invasiveness of HCC (Nakanishi et al., 2005). In addition, Ghalali and his team confirmed that Atto vastatin can reduce hepatitis B virus X protein-(HBx) and insulin-induced PACT and pGsk3 β levels, and antagonize Akt-induced adipogenesis, but these effects depend on P2XR(s). Therefore, we concluded that the imbalance of AKT pathway may promote the development of hepatocellular carcinoma, and P2X-AKT signal pathway may play an important role in the anti-cancer effect of statins (Ghalali et al., 2017).

## Pancreas

The pancreas is a complex glandular organ with exocrine and endocrine. Some studies have confirmed that P2XR(s) are widely expressed in the pancreas, mainly expressed in the pancreatic duct cells, islet beta 1 cells, HIT cells, islet Beta-TC6 cells, pancreatic stellate cells, and pancreatic ductal adenocarcinoma cell line. It also plays an important role in exocrine, endocrine, β-cell apoptosis, and sensory signal transmission in the pancreas (Lynch et al., 1999; Coutinho-Silva et al., 2001; Ohtani et al., 2011). First of all, a number of experimental studies have confirmed that P2X7R is expressed in the pancreatic duct, which can induce the increase of intracellular Ca^2+^ concentration under the action of ATP (Christoffersen et al., 1998; Wang et al., 2013). Knockout of pancreatic P2X7 receptors leads to a decrease in Ca^2+^ signal, which eventually leads to a significant decrease in pancreatic ductal secretion; however, there is a gender difference in this mode of induction (Novak et al., 2010). In terms of pancreatic endocrine regulation, P2X3, P2X4, and P2X7 receptor have been identified in INS-1β cells, mouse, rat, and human pancreatic β cells, and it has been confirmed that p2XR(s) in pancreatic β cells are involved in insulin secretion, glucose metabolism, and β-cell apoptosis (Lynch et al., 1999; Coutinho-Silva et al., 2001; Ohtani et al., 2011). It has been found that insulin release is triggered by intracellular Ca^2+^ concentration and has a certain glucose concentration dependence. The increase of Ca^2+^ concentration in cells can trigger insulin secretion vesicles containing insulin to release insulin through exocytosis, and extracellular ATP activates P2X receptor to open the receptor ion channel pore to mediate Ca^2+^ influx, thus regulating insulin release (Burnstock, 2014). In the report of Geschwind and his team, hamster cloned β-cell line HIT cells are selected to confirm that ATP stimulated Ca^2+^ influx rather than Ca^2+^ storage and release, suggesting that the receptor manipulated the channel may be P2XR(s) (Geschwind et al., 1989). It has been reported that P2XR(s) in rat β cells are activated by ATP or P2XR(s) agonist α, β-meATP at low or non-stimulating glucose concentration, which induces transient insulin release (Coutinho-Silva et al., 2001; Burnstock, 2014). In human β cells, ATP activates ionic P2X3R in the plasma membrane of β cells, opening the pores of P2X3R ion channels, resulting in the influx of Ca^2+^/Na^+^, resulting in membrane depolarization, and action potential frequency increasing the Ca^2+^ flux through your high voltage-gated Ca^2+^ channels, thereby stimulating insulin release. At the same time, insulin vesicles also contain a certain amount of ATP, so the simultaneous release of ATP and insulin and the activation of P2XR(s) amplify glucose-induced insulin release, thus making the secretion mechanism of β cells more sensitive (Jacques-Silva et al., 2010). Similarly, it has been confirmed that P2X4R is also involved in the regulation of insulin secretion in mouse islet Beta-TC6 cells and is related to the regulation of islet cell proliferation and cell viability (Ohtani et al., 2011). The study also found that P2X7R regulates the quality of islet cells by regulating the secretion of IL-1 and is related to β-cell apoptosis in the later stage of type 2 diabetes (Giannuzzo et al., 2015). So far, many studies have reported that P2XR(s) play an important role in acute pancreatitis, chronic pancreatitis, diabetes, and pancreatic cancer.

### P2X Receptors and Acute Pancreatitis

Acute pancreatitis (AP) is a kind of aseptic inflammation with high annual incidence, and about 20% of patients develop into severe Acute pancreatitis (SAP), with high mortality. It is characterized by early activation of intracellular proteases followed by acinar cell death and inflammation. In murine acute pancreatitis induced by Hoque and his colleagues with caerulein, TLR9 and P2X7R are important DAMP receptors upstream activated by inflammatory bodies. The use of P2X7R antagonist AME 439079 can reduce the pancreatic edema and significantly reduce pancreatic leukocyte infiltration (Hoque et al., 2011). In addition, Zhang and his team confirmed that the emodin reduces the concentration of IL-1β and IL-18 in plasma by inhibiting P2X7R/NLRP3 signal pathway in rats with acute pancreatitis, thus limiting the progress of SAP (Zhang et al., 2019).

### P2X Receptors and Chronic Pancreatitis

It was reported as early as 1995 that inflammatory cell infiltration, progressive organ atrophy, and disorder of collagen deposition occurred in chronic pancreatitis (Steer et al., 1995). Extracellular nucleotides are regarded as important mediators of inflammation in many pathological situations. Künzli and his team propose the role of purinergic signal transduction in the chronic pancreatitis; their study confirmed a significant increase in transcripts of exonucleases and P2R (P2X7R, P2Y2R, and P2Y6R) in chronic pancreatitis and is associated with chronic inflammation and neoplasia of the pancreas (Künzli et al., 2007). As is known to all, chronic pancreatitis results in organ fibrosis, pain, and exocrine and endocrine insufficiency. The formation of fibrosis and pain are important characteristics of chronic pancreatitis, activation of pancreatic stellate cells (PSC) plays a key role in pancreatic fibrosis. Inflammation and repetitive pancreatic injury lead to cytokine-mediated activation and oxidative damage to cells, with the potential to increase extracellular nucleotide release and accumulation, extracellular nucleotides regulate inflammation, and immunity *via* purinergic/pyrimidinergic P2-receptors (P2XR and P2YR), extracellular nucleotides are hydrolyzed by ecto-enzymes, such as nucleoside triphosphate diphosphohydrolase-1 (CD39/NTPDase-1; Dranoff et al., 2002). It is reported that PSC expresses P2X7R at mRNA transcripts and protein levels. CD39 deletion decreases fibrogenesis in experimental pancreatitis. Therefore, extracellular nucleotides and P2X7R are important regulators of the PSC proliferation and death (Künzli et al., 2008; Haanes et al., 2012). In addition, p2XR(s) are associated with the pain mechanism of chronic pancreatitis. In the rat, model of chronic pancreatitis induced by trinitrobenzene sulfonic acid (TNBS). TNBS injection produced a significant upregulation of P2X3R expression and an increase in ATP-evoked responses of pancreatic DRG neurons. Pancreatic hyperalgesia is markedly attenuated by administration of purinergic receptor antagonist suramin (Wang et al., 2015).

### P2X Receptors and Diabetes

Interestingly, P2XR(s) are also involved in mediating insulin secretion, survival, and apoptosis of islet β cells, indicating that P2XR(s) also play a role in diabetes. We all know that type 1 and type 2 diabetes are inflammatory diseases. P2X7R knockout mice can prevent type 1 diabetes induced by streptozotocin and limit the increase in levels of pro-inflammatory mediators (IL-1β, interferon-γ, and nitric oxide; Vieira et al., 2016). In STZ-induced diabetic rats, the P2X7R on the glucagon-containing alpha cells in the islets is increased, and they migrate in the center to replace the missing insulin-containing β cells (Coutinho-Silva et al., 2003). Other studies have shown that P2X7R inhibition can be used as an adjuvant therapy to delay the progression of diabetic nephropathy (Rodrigues et al., 2014).

### P2X Receptors and Pancreatic Cancer

In recent years, P2XR(s) have also been reported in pancreatic cancer, and it has been found that the expression of P2X7R is upregulated in pancreatic cancer and chronic pancreatitis (Künzli et al., 2007). In 2015, Giannuzzo and his team confirmed that P2X7 is overexpressed in human pancreatic ductal adenocarcinoma cell line (PDAC), and regulated the survival, migration, and invasion of pancreatic ductal adenocarcinoma cells, in which P2X7R had different effects on PDAC cell survival. Specific P2X7R’S agonist BzATP had cytotoxic effect on all cell lines and mediated cell necrosis, and P2X7R pore antagonist A438079 inhibited the proliferation of all PDAC cell lines (Giannuzzo et al., 2015, 2016). In the treatment of pancreatic cancer, some studies have reported the progress of statins in inhibiting PDAC by targeting the P2X7-Akt axis or P3IK/Akt signal and making pancreatic cancer cells sensitive to chemotherapeutic drugs (Mistafa and Stenius, 2009; Mohammed et al., 2012).

## Intestine

In the gutl tract, P2XR(s) are mainly distribute in the submucosal nerve plexus of the intestinal tract, myenteric nerve plexus, both longitudinal and circular muscle of the colon, intestinal epithelial cancer cells (HCT8 and Caco-2), and colorectal cancer cells. There are also differences in the expression of the P2XR(s) subtype, in which p2X2R, P2X3R, P2X5R, and P2X6R are express in myenteric plexus and Submucosal plexus. P2X6R mainly expressed on intrinsic sensory neurons, such as in the large size neurons which resembled Dogiel type II neurons and NeuN-ir cells, may be involved in regulating the physiological functions of these neurons. In addition, P2X4R expressed in macrophages of the rat gastrointestinal tract and p2X7R mainly expressed in colorectal cancer cells. It was found that P2X2R is immunoreactive in the intermuscular neurons of the longitudinal and circular muscles of the colon, which are mainly involved in mediating the contraction of colonic muscle (Giaroni et al., 2002). Therefore, P2XR plays an important role in intestinal diseases, such as intestinal hypersensitivity, inflammatory bowel disease, intestinal transport disorders, and intestinal tumors.

### P2X Receptors and IBS

Irritable bowel syndrome (IBS) is a functional bowel disorder that presents with no structural and biochemical abnormalities, and it is clinically characterized by abdominal pain, abdominal distention, alterations in bowel habits, and changes in stool characteristics. The present pathogenesis of IBS remains unclear. The most important mechanisms recently implicated in the genesis of IBS symptoms are the abnormal intestinal motility, changes in the function of the sensory, and the enhanced intestinal nociception. According to the report that p2XR(S) are distributed on subsets of myenteric and submucous neurons of the ENS, glia, ICC, smooth muscle, epithelia and EC cells, and immunochemical. Electrophysiological and calcium imaging studies confirmed the role of P2X(s) ion channel receptors in excitatory neurotransmission and information transfer between neurons and glia cells. ATP is a danger signal molecule in the gastrointestinal tract and its release is likely mediated from both local epithelial cells and nerves to modulate peristalsis, secretion, and nociception. Early studies have shown that in the process of visceral hyperalgesia and visceral hypersensitivity induced by IBS in rats, colonic dilated intestinal epithelial cells release ATP to activate P2XR(s) (P2X3R) innervating colonic afferent nerve endings, which mediates mechanical sensory transduction, such as visceral pain and hypersensitivity (Wynn et al., 2003; Xu et al., 2008). In a model without colonic inflammation, Shinoda and his team have shown that P2X3R mediates different colonic mechanosensitivity and colon zymosan hypersensitivity in mice (Shinoda et al., 2009). In addition, in the rat model of diabetic colonic hypersensitivity induced by streptozotocin (STZ), it was found that the excitability and P2X3R expression of rat colon-specific dorsal root ganglion (DRG) neurons are increased, and this effect could be inhibited by lipoic acid (ALA), which provides a new therapeutic target for diabetic colonic hypersensitivity (Hu et al., 2017). AIEC LF82 infection can increase the expression of P2X4R and P2X7R in CEABAC10 hypersensitive mice. P2X3R exists in the sensory neurons of the central nervous system and the peripheral nervous system, is activated by low levels of ATP, and can be sensitized after inflammation or nerve injury (Lashermes et al., 2018). Therefore, P2XR(s) antagonists are of great significance in the treatment of IBS. Due to pain, diarrhea, constipation, and pain are the characteristic symptoms of IBS, so it is necessary to target and antagonize these symptoms, respectively. Relevant literature shows that P2X2/3R, P2X3R, and P2X7R antagonists are potential drugs for visceral pain and that includes A-317491, AF-353, and TNP-ATP. Several diaminopyridines were shown to be selective antagonists at P2X3R and P2X2/3R, and had *in vivo* efficacy in a pain model (Ballini et al., 2011). For diarrhea and constipation, agonists acting at enteric P2XR(s) may enhance gastrointestinal propulsion and secretion, and these drugs could be useful for treating constipation-predominant IBS and antagonists acting at enteric P2XR(s) would decrease propulsion and secretion and they might be useful for treating diarrhea-predominant IBS (Galligan, 2004).

### P2X Receptors and IBD

According to the related literature, the role of P2XR(s) has been explored in the pathological mechanism of IBD. Inflammatory bowel disease (IBD) is a kind of chronic intestinal disease, which mainly includes Crohn’s disease (CD) and ulcerative colitis (UC). In recent years, some teams have proposed that the overactivation of intestinal immune cells (including macrophages and T helper cells) promotes the progress of IBD in the pathogenesis of IBD (Brown and Mayer, 2007). Interestingly, a large amount of extracellular ATP produced by inflammatory injury promotes immune response and inflammatory response by activating P2XR(s). The expression of P2X3R is increased in the myenteric plexus of human colitis and is significantly upregulated in inflammation and hypersensitivity, suggesting that it plays a role in dyskinesia and pain (Yiangou et al., 2001; Wynn et al., 2004). In the report of Zoetewij and his team, it was pointed out that in the rat colitis model induced by sodium dextran sulfate (DSS), P2XR(s)’s antagonist blocked ATP-P2X7R signal transduction and inhibited the activation of NF κ B and the expression of caspase-1 in lamina propria immune cells, thus inhibiting the levels of TNF and IL-1β. Therefore, antagonistic P2X7R can limit the development of IBD. However, Hofman and his team suggest that in the treatment of IBD with P2X7 receptor antagonists, it limits intestinal inflammation and promotes the proliferation of intestinal epithelial cells, which protects intestinal epithelial cells from apoptosis, besides, it increases the risk of colitis-associated cancer (CAC; Hofman et al., 2015). Both inflammatory bowel disease (IBD) and irritable bowel syndrome (IBS) are characterized by visceral abdominal pain with colonic hypersensitivity (CHS). Some studies have shown that P2X3R is not involved in sensory transmission under physiological conditions but is mainly transduced in the intracellular environment through extracellular ATP binding to P2X3R signal in the late stage of acute TNBS colitis and inflammation, including Cdk5. Csk and CASK kinases regulate colonic hypersensitivity, suggesting that P2X3R is a potential new target for the treatment of abdominal pain syndrome (Volonté and Burnstock, 2013; Deiteren et al., 2015). In the Lashermes team study, transgenic mice expressing intestinal CEACAM6 receptors were used to study whether AIEC LF82 infection led to the development of colonic hypersensitivity. It was found that colonic hypersensitivity induced by AIEC LF82 bacteria is associated with increased expression of P2X3R, P2X4R, and P2X7R (Lashermes et al., 2018). Therefore, knocking out or antagonizing P2X7 receptor is a new target for the treatment of IBD. Research shows that AZD9056 is an adamantane amide and selective P2X7R antagonist, being evaluated for safety and efficacy in causing clinical remission in CD patients – This is the first clinical trial in IBD patients with a P2X antagonist. Additional phase I and II clinical trials are currently underway regarding the safety and efficacy of P2X7R antagonists. It is believed that P2XR(s) antagonists will become an important target of IBD in the near future.

### P2X Receptor and Intestinal Cancer

In recent years, it has been found that P2XR(s) also play an important role in intestinal tumors. P2XR(s) participate in the regulation of proliferation and apoptosis of human intestinal epithelial cancer cells (HCT8 and Caco-2), and the action of high concentration of ATP activates P2X7R and induces cancer cell apoptosis (Coutinho-Silva et al., 2005). Some studies have shown that compared with normal tissues, P2X7R is overexpressed in colorectal cancer cells. Overexpressed P2X7R is closely related to tumor size, metastasis, and TNM stage of colorectal cancer. The expression of P2X7R may be a potential biomarker for judging prognosis and metastasis of colorectal cancer (Qian et al., 2017). In 2020, Zhang and his team confirmed that P2X7R is involved in the activation of STAT3 signal and the regulation of EMT-related gene expression in colon cancer cells induced by ATP, and the activation of P2X7R can also stimulate STAT3 pathway and regulate the expression of MMP-2 and E-cadherin. These genes are the key factors of tumor cell migration and invasion, so we conclude that P2X7R promotes the invasion and migration of colon cancer. This may be an important potential target for the treatment of colon cancer (Zhang et al., 2020).

## Conclusion

Undoubtedly, as research on P2XR(s) continues, the possibility of P2XR(s) become a therapeutic target for the treatment of diseases is advanced. Similarly, the physiology, pathology, and mechanism of P2XR(s) in digestive system have been revealed constantly. However, there are still some mechanisms that have not been fully clarified and even controversial. P2XR(s), as an ionic channel receptor, are mainly activated directly or indirectly by ATP as the main ligand, resulting in cell membrane depolarization, triggering action potentials, and transmitting a series of sensory signals to the nerve center, such as in the regulation of chronic visceral pain. P2X3R and P2X2/3R expressed in the primary sensory afferent nerves of the visceral vagus nerve can be activated by ATP to directly sensitize C fibers through membrane depolarization and calcium entry, thus promoting the transmission of pain signals to the center. What is more, some of these subtypes (such as P2X7R) can activate a series of signaling pathways in cells, leading to a wide range of cellular responses, thus promoting the regulation of corresponding physiological and pathological functions. For example, under the condition of liver injury or infection, it can induce cells to release ATP molecules and activate P2X7R on liver immune cells to induce a large number of K^+^ influx to activate the release of inflammatory corpuscles and process inflammatory cytokines which can cause liver inflammatory lesions, such as alcoholic hepatitis, liver fibrosis, and so on. In addition, in recent years, a large number of studies have confirmed that P2XR(s) play a key role in the invasion, metastasis, proliferation, and apoptosis of digestive system tumors, especially P2X7R has become a potential biomarker of prognosis and metastasis of many digestive system tumors and is closely related to cancer pain. Therefore, the development of P2XR(s) blockers can not only reduce cancer-related pain, but also delay tumor progression. It is believed that P2XR(s) may be a key therapeutic target for many digestive diseases in the future with increasing studies on the pharmacology of P2XR(s) in the future.

## Author Contributions

QA and GY equally contributed to this study and wrote the manuscript. XY, JL, WS, YH, QD, and QL collected the literature. JX primarily revised and finalized the manuscript. RX revised the manuscript for clarity and style. All authors have read and approved the final manuscript. RX and JX are the co-corresponding authors.

## Funding

This study was supported by research grants the National Natural Science Foundation of China (nos. 81660099, 82170628, 81970541, 31960151, and 32160208) and Collaborative Innovation Center of Chinese Ministry of Education (2020-39) and the Graduate Research Fund Project of Guizhou Province [nos. YJSCXJH(2020)171 and YJSCXJH(2020)166].

## Conflict of Interest

The authors declare that the research was conducted in the absence of any commercial or financial relationships that could be construed as a potential conflict of interest.

## Publisher’s Note

All claims expressed in this article are solely those of the authors and do not necessarily represent those of their affiliated organizations, or those of the publisher, the editors and the reviewers. Any product that may be evaluated in this article, or claim that may be made by its manufacturer, is not guaranteed or endorsed by the publisher.

## Abbreviations

R: receptor
BzATP: dibenzoyl-ATP
TM: transmembrane
DRG: dorsal root ganglia
α,β-meATP: α,β-methylene ATP
NG: nodular ganglion
IGLEs: Intraganglionic laminar endings
APAP: acetaminophen
A-438079: P2X7 receptor antagonist
AP: acute pancreatitis
BBG: P2X7 receptor antagonist brilliant blue
TAA: thioacetamide
PSC: pancreatic stellate cells
